# A mathematical model of insulin action on acinar tissue linking histology and radiological imaging of the pancreas

**DOI:** 10.3389/fendo.2025.1676627

**Published:** 2025-09-16

**Authors:** John Virostko

**Affiliations:** ^1^ Department of Diagnostic Medicine, Dell Medical School, University of Texas at Austin, Austin, TX, United States; ^2^ Oden Institute for Computational Engineering and Sciences, The University of Texas at Austin, Austin, TX, United States

**Keywords:** diabetes, type 1 diabetes, volume, peri-insular, islets, endocrine, exocrine

## Abstract

The pancreas is smaller in individuals with type 1 and type 2 diabetes. The etiology of this reduced pancreatic volume is not fully understood, but it may be due to loss of insulin’s trophic influence on exocrine pancreatic tissue. Supporting this, histological studies have identified a zone of acinar cell hypertrophy and hyperplasia surrounding pancreatic islets, putatively due to insulin action on peri-islet acinar tissue. This study develops a mathematical model of pancreas size incorporating beta cell density, beta cell clustering, and the magnitude and spatial extent of acinar cell expansion to estimate the relationship between beta cell mass and pancreas size. This model indicates that growth of acinar tissue surrounding the beta cell is sufficient to account for the smaller pancreas volume observed in individuals with diabetes. Furthermore, single beta cells and smaller beta cell clusters have a greater influence on pancreas size on a per cell basis, as larger islets have greater overlap in the zone of insulin action. Thus, changes in pancreas volume may be more sensitive to loss of single beta cells or small islets than larger islets. The model provides a conceptual framework linking histological and radiological imaging to better understand the relationship between pancreas volume and beta cell mass.

## Introduction

Pancreas size is smaller in individuals with type 1 (T1D) and, to a lesser extent, type 2 diabetes (T2D) (1). Moreover, pancreas size is dynamic, declining as individuals progress through presymptomatic stages of T1D (2) and increasing in individuals with T2D remission (3). Pancreas size also changes over the lifespan with growth during childhood and adolescence and decline in older age (4). Islet transplantation studies indicate that the size of the pancreas size is proportional to the number of isolated islets (5), although other studies have found mixed results (6). Collectively, these results suggest that pancreas size correlates with beta cell mass and/or function, although the precise nature of this correlation remains unknown. Furthermore, the extent of pancreas size reduction in T1D (30–40% smaller) (1) surpasses the small fraction of the pancreas made up of beta cells (1-2%) (7), implicating loss of acinar tissue.

While a smaller pancreas size in individuals with T1D was observed 8 decades prior (8), the implications of this smaller pancreas size have not been well characterized. Furthermore, unlike the pancreatic beta cells, which pose significant challenges for imaging due to their small size and scattered distribution throughout the pancreas, pancreas size can be readily assessed with magnetic resonance imaging (MRI) or computed tomography (CT). Radiological imaging of the pancreas may provide information about diabetes progression or therapeutic response not captured by blood tests (2). However, the relationship between pancreas size and beta cell mass are not well understood. For example, it is not known whether there is a linear relationship between beta cell mass and pancreas size or whether there is a threshold of beta cell loss needed to detect changes in pancreas size.

Two mechanisms have been postulated to link beta cell loss and acinar cell atrophy: loss of trophic effects of insulin and autoimmune destruction of acinar tissue. Supporting the former, pancreas size in individuals with insulin deficiency, but lacking autoimmunity, have similar pancreas size as T1D (9). Additionally, acinar cell hypertrophy is observed in peri-islet regions of db/db mice (10), while chemical destruction of the beta cell eliminates peri-islet acinar hypertrophy (11). Human acinar tissue exhibits limited hypertrophy surrounding islets, but increased proliferation, suggesting that hyperplasia may be more dominant in humans (12). Together, these findings suggest absence of insulin may be a primary driver of pancreas atrophy in diabetes.

There are no studies that connect structural insights from pancreas autopsy studies with imaging observations of the pancreas in individuals with diabetes. Imaging of the entire intact excised human pancreas can offer a critical bridge between high-resolution histology and whole-organ radiological imaging (13). This study builds a mathematical model of pancreas size incorporating beta cell density and clustering estimates from excised pancreas studies to evaluate the effect of acinar tissue expansion on radiological imaging. This model can be used to guide interpretation of pancreas size measures and estimate relative loss of beta cell mass in individuals with diabetes.

## Research design and methods

All statistical analyses and mathematical modeling were performed using R (version 4.3.1) within the RStudio environment (Posit, Boston, MA). Custom scripts were developed to implement the modeling framework, and standard packages were used for data processing, visualization, and statistical inference.

### Geometric representation of beta cell distribution and pancreas volume

We modeled each beta cell as a sphere with radius of 5 µm (diameter of 10 µm), in agreement with prior histology studies (14, 15). Equation 1 was used to calculate the volume of each beta cell:


(1) $$Volume_{beta\;Cell}=\frac{4}{3}\;\pi r_{beta\;cell}^{3}$$

This yields a volume for a single beta cell of 524 µm^3^, in agreement with prior reports (16).

Islets were modeled as clusters of a range of number of beta cells, reflecting growing realization that islet sizes are more diverse than previously appreciated as our tools for imaging these small cell clusters improves (13). We modeled clusters consisting of a single beta cell, 5 cells, 25 cells, 100 cells, 250 cells, and 1000 cells (17). Each cell cluster was modeled as a perfect sphere with the same volume as the sum of the individual cells packed together. This assumes no extracellular space of packing inefficiencies. In addition to analyses of single-sized cell clusters, we modeled a distribution of beta cell cluster sizes throughout the pancreas, approximating findings from autopsy studies (13).

For beta cell density, we modeled a maximum beta cell volume comprising 3% of the pancreas based on a recent study (13) updating prior estimates of 1-2% beta cell density. We assumed that individuals with longstanding T1D have a complete loss of beta cells, in accordance with a prior report of 99% beta cell loss (18), although we note other studies have found a small population of beta cells remaining in T1D (19). Accordingly, we note that all results display beta cell densities ranging from 0-3%, and thus can be used to estimate changes in beta cell mass spanning this range. Pancreas size is influenced by body size, age, and sex, leading to a range of differences in pancreas size between non-diabetic controls and individuals with T1D (1). For our model, we assumed a non-diabetic pancreas volume of 90ml and a pancreas volume of 60ml in individuals devoid of beta cells, derived by averaging published results from a meta-analysis reporting pancreas volume in controls and individuals with longstanding T1D (1).

### Modeling insulin action

**Figure 1:**
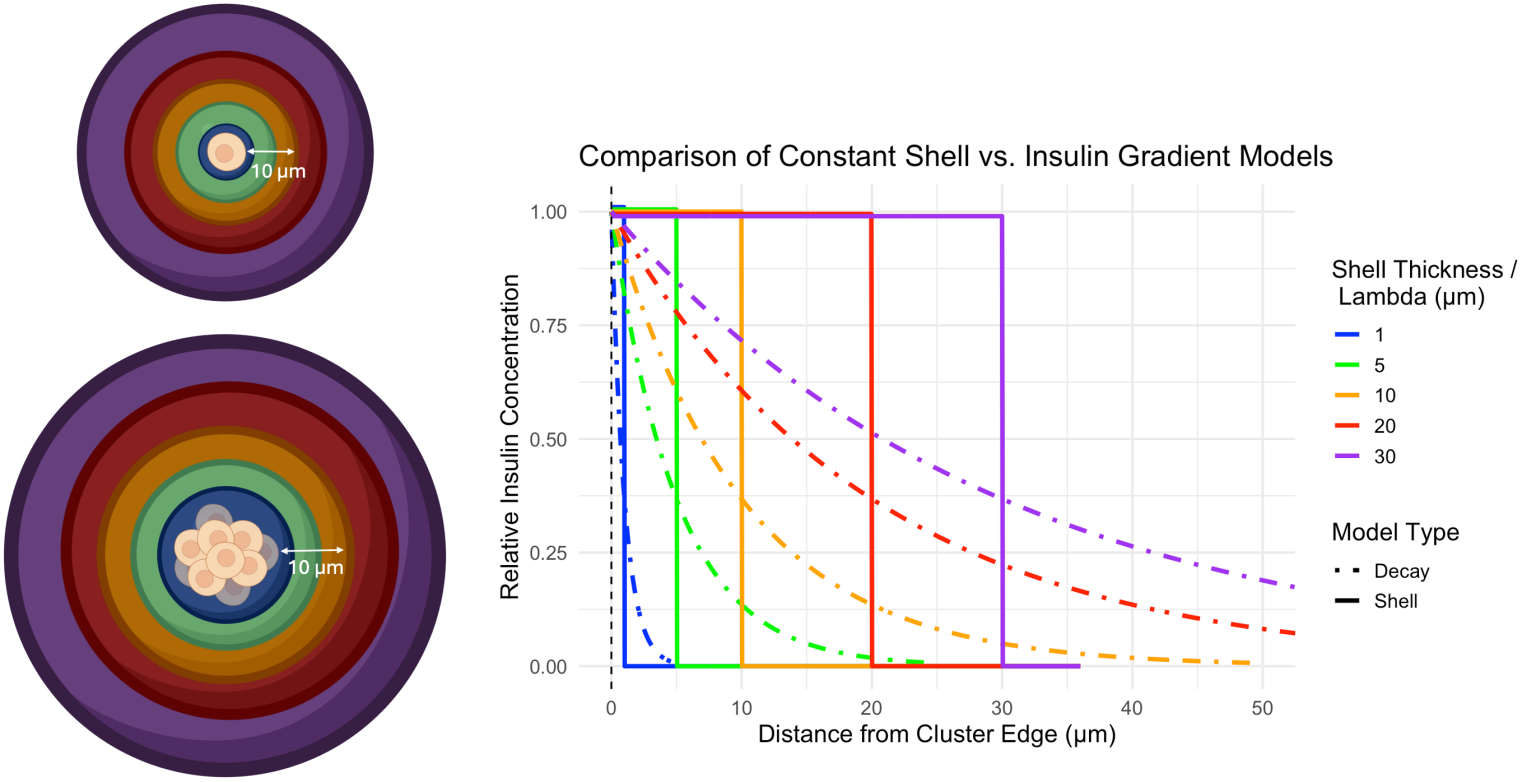
Left) Cartoon depicting the influence of a beta cell on a concentric shell of acinar tissue surrounding a single beta cell (top) or cluster of beta cells (bottom). A shell of thickness 10 µm is shown for illustrative purposes. Right) Graph of insulin action on surrounding acinar tissue as a function of distance from the cell cluster edge. Colors display various distances from the beta cell cluster upon which insulin drives acinar cell growth and correspond with the colors in the cartoon. Two models of insulin action are displayed. The constant shell model (solid line) assumes insulin acts homogenously within a shell surrounding the beta cell cluster of prescribed thickness. The insulin gradient model (dashed line) assumes insulin action is strongest at the outer edge of the beta cell cluster and decays exponentially with increasing distance from the beta cell cluster. The decay length parameter models how quickly the concentration of insulin decreases as you move away from a beta cell cluster (lower means faster decay). Cartoon was created using https://BioRender.com.


We modeled insulin action as a concentric sphere surrounding the beta cell (or cluster of beta cells) as displayed in **Figure 1**. In the first approach, termed the constant shell model, we assumed that the trophic effect of insulin acted homogenously within a sphere surrounding the beta cell cluster (**Figure 1**). The equation describing this shell is given in Equation 2:

(2) $$Volume_{Constant\;Shell}=\frac{4}{3}\;\pi r_{shell}^{3}-\frac{4}{3}\;\pi r_{beta\;cell}^{3}$$

The thickness of this shell, r_shell_, was modeled as 1, 5, 10, 20, and 30 µm beyond the outer edge of the beta cell cluster, building upon a recent report using a 33 µm width for peri-islet insulin action (20).

In a second approach, termed the insulin gradient model, we assumed that insulin concentration was highest at the outer edge of the beta cell cluster and decayed exponentially with increasing distance from the beta cell cluster. This model includes a decay length parameter ( λ ) that represents how quickly the concentration of insulin decreases as you move away from a beta cell cluster. The volume of this insulin gradient model is given in Equation 3:


(3) $$Volume_{Insulin\;Gradient}=\int_{r_{beta\;cell}}^{r_{beta\;cell}+5\lambda }4\;\pi r^{2}\cdot e^{-(r-r_{beta\;cell})/\lambda }dr$$

The decay length, λ , was set at 1, 5, 10, 20, and 30 µm to determine the distance over which insulin has a significant effect on the surrounding tissue. A small decay length means insulin’s influence drops off rapidly and is limited to areas very close to the cluster, while a larger value means insulin spreads out further, affecting a wider region around the cluster. A plot of the effect of insulin concentration as a function of decay length and distance from the beta cell cluster edge is shown in **Figure 1**. For these initial analyses, we assumed that the acinar tissue volume doubled within the volume surrounding the beta cell cluster.

To simulate varying degrees of acinar cell expansion around beta cells, we adjusted the magnitude of acinar cell growth within the shell surrounding the beta cell cluster. The maximum expansion was set to 100%, corresponding to the doubling of acinar cell volume in the affected volume used in prior analyses. Expansion was also modeled as 50% and 20% increases to simulate smaller amounts of acinar tissue growth in response to insulin. These varying levels of expansion allowed us to examine how different degrees of acinar cell growth might influence pancreas size.

## Results

**Figure 2:**
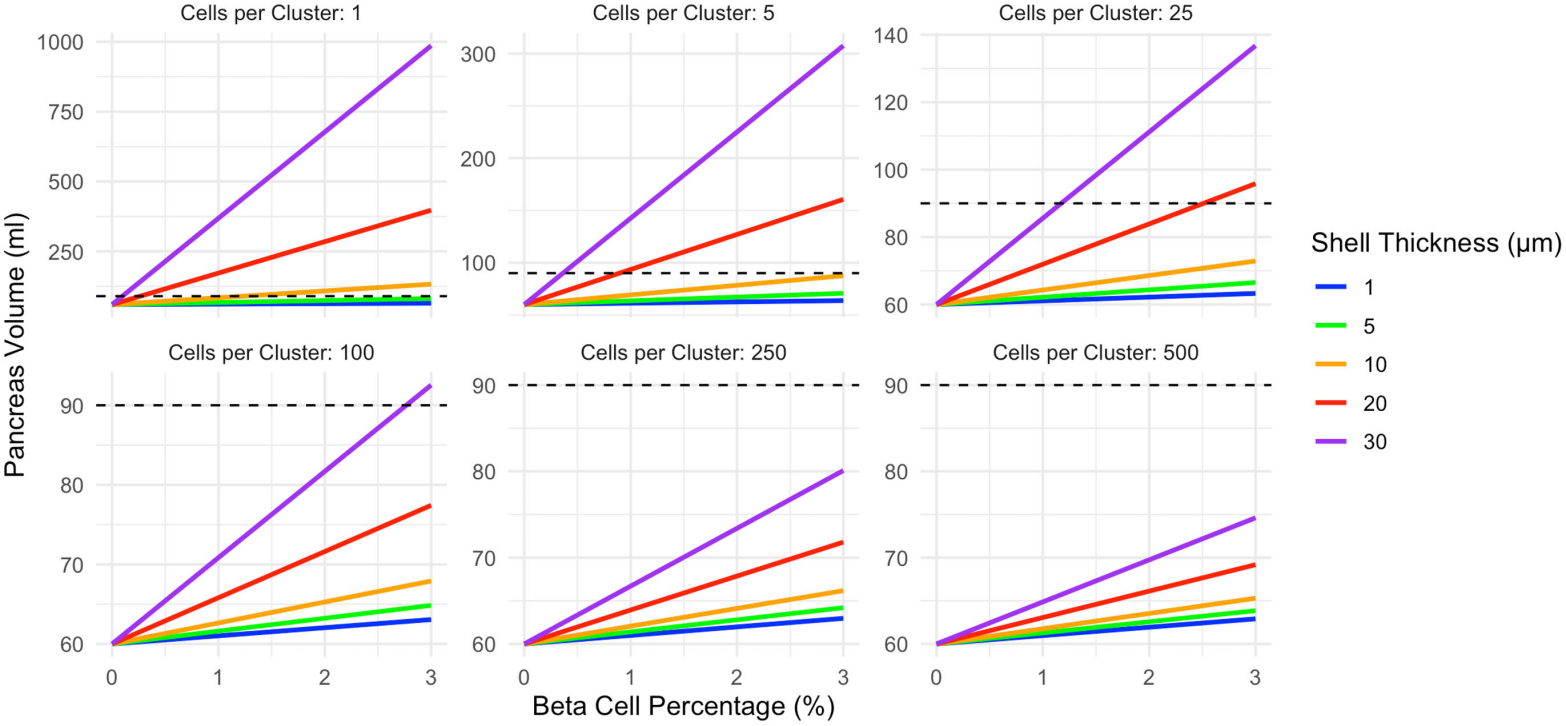
In the constant shell model, pancreas volume correlates with beta cell density. The slope of this relationship is influenced by both the beta cell cluster size (shown in different panels) and the thickness of acinar tissue expansion surrounding each cell cluster (displayed according to color). Smaller beta cell clusters lead to larger pancreas volume due to diminished overlap in the shells surrounding each cluster. The pancreas volume of a non-diabetic control (90ml) is shown as a dashed line. This model assumes acinar tissue doubles within the affected volume. Note the different y-axis maximum in each panel.

The relationship between beta cell mass and pancreas size depended on the spatial extent of insulin action as well as the beta cell cluster size. In the constant shell model, a wider thickness of affected acinar tissue increased the slope of the line linking beta cell density and pancreas volume (**Figure 2**). Smaller clusters of beta cells resulted in higher pancreas volumes for a given beta cell density (**Figure 2**). Note the different y-axis in panels of **Figure 2**. This relationship between cell clustering and pancreas size results from greater overlap in the shells surrounding larger clusters of beta cells versus smaller clusters of individual beta cells. For example, a single beta cell with an influence radius of 20 µm leads to an increased acinar tissue volume of 64,973 µm^3^. 100 of these single beta cells with no overlap would lead to an increased volume of 6,497,300 µm^3^. In contrast, a cluster of 100 beta cells would only lead to an increased volume of 287,431 µm^3^, 22 times smaller, due to overlap of the sphere of influence of these 100 clustered cells. The dashed lines on **Figure 2** display a pancreas volume of 90ml, the estimated volume of a pancreas with a full complement of beta cells. For single beta cell clusters, shell thicknesses of 10, 20, or 30 µm exceed the 90ml pancreas volume at 3% beta cell density. Clusters of 250 or 500 beta cells do not reach 90ml at any shell thickness modeled.

**Figure 3:**
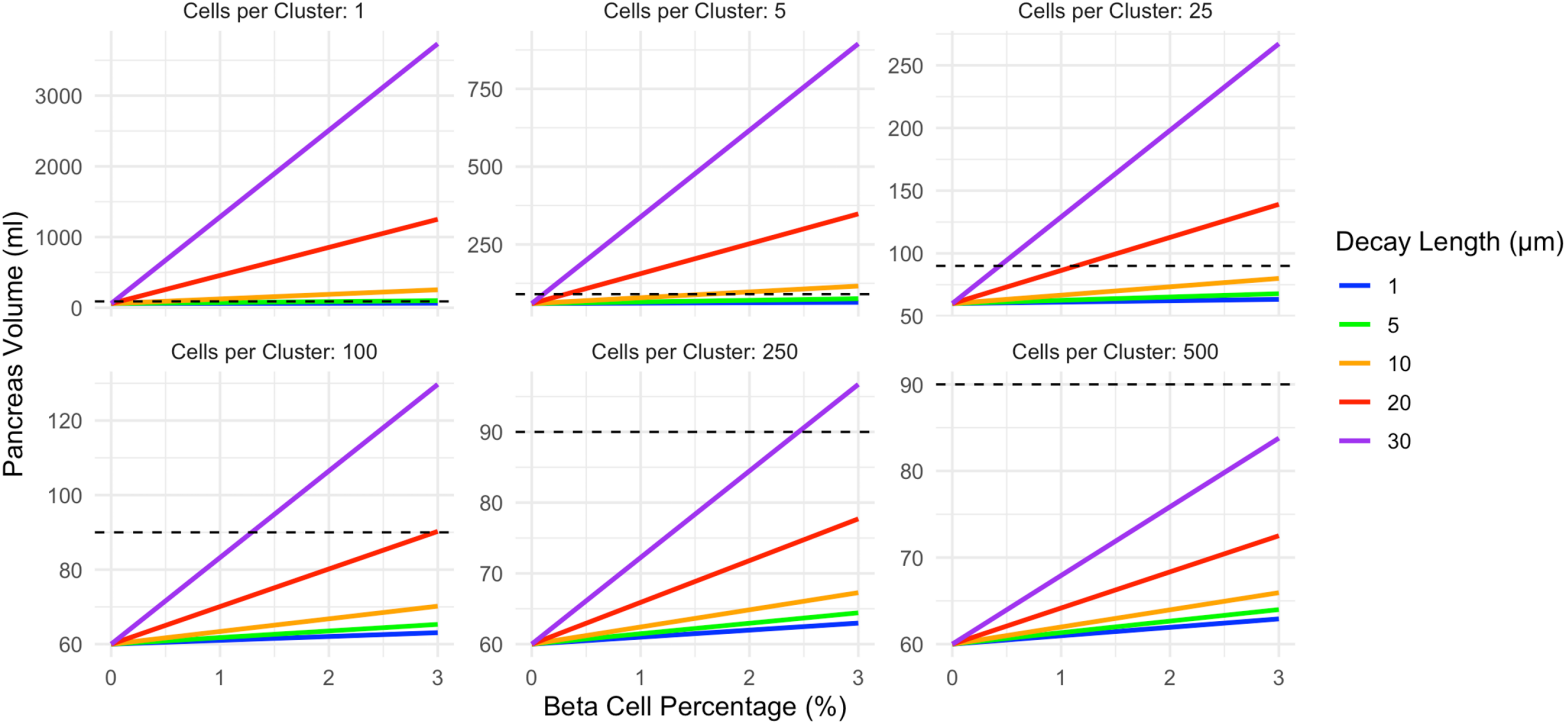
In the exponential decay model, pancreas volume correlates with beta cell density and is higher than the corresponding shell model. The slope of this relationship is influenced by both the beta cell cluster size (shown in different panels) and the decay length of acinar tissue expansion surrounding each cell cluster (displayed according to color). Increasing decay length corresponds to a larger volume of acinar tissue expansion, and more strongly influences the decay model than the constant shell model. The pancreas volume of a non-diabetic control (90ml) is shown as a dashed line. This model assumes acinar tissue doubles within the affected volume. Note the different y-axis maximum in each panel.

In the insulin gradient model (**Figure 3**), pancreas volume is higher than the corresponding shell model for a given beta cell cluster size. The correlation between beta cell density and pancreas volume is influenced by both the beta cell cluster size and the decay length of acinar tissue expansion surrounding each cell cluster. Again, note the different y-axis in panels of **Figure 3**. Increasing decay length corresponds to a larger volume of acinar tissue expansion. For single beta cell clusters, all decay lengths can exceed the 90ml pancreas volume at 3% beta cell density. For beta cell clusters of 250 only a 30 µm decay length exceeds the 90ml pancreas volume at 3% beta cell density. Clusters of 500 beta cells cannot reach a pancreas volume of 90ml at any decay length modeled.

**Figure 4:**
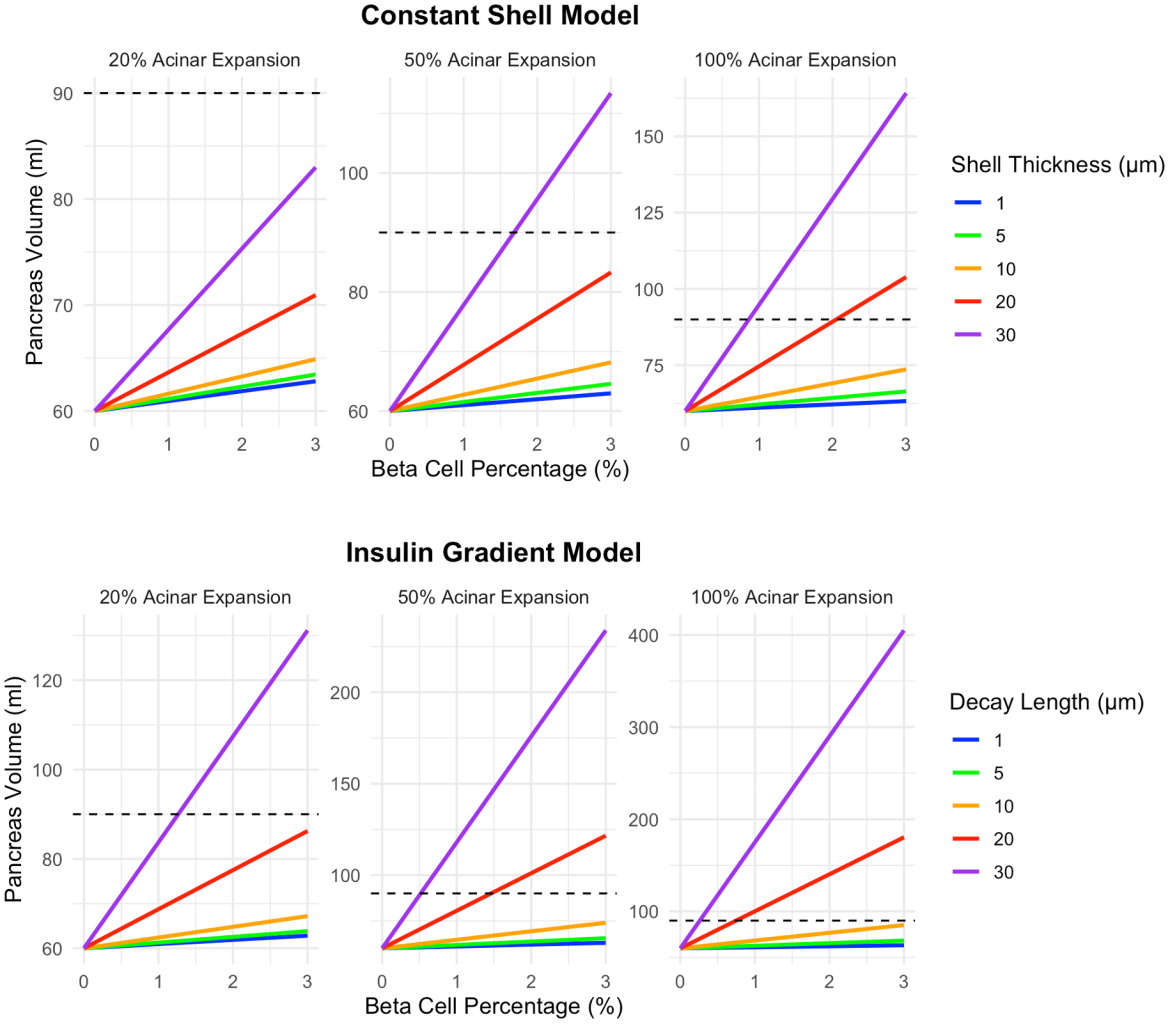
A higher magnitude of acinar tissue expansion surrounding beta cell clusters leads to increased pancreas volume. Graphs illustrate increases of 20%, 50%, and 100% (a doubling of acinar tissue volume within the volume surrounding the beta cell cluster). The top row displays results for the shell model while the bottom row shows results for the exponential decay model. This model assumes a heterogeneous distribution of 5% single beta cells, 10% clusters of 10 cells, 20% clusters of 25 cells, 40% clusters of 100 cells, 20% clusters of 250 cells, and 5% clusters of 1000 cells. Note the different y-axis maximum in each panel.

Given the strong influence of beta cell cluster size on pancreas volume, and the existence of variable islet sizes within the pancreas, we modeled further results as a distribution of beta cell cluster sizes. Specifically, we modeled a distribution of 5% single beta cells, 10% clusters of 10 cells, 20% clusters of 25 cells, 40% clusters of 100 cells, 20% clusters of 250 cells, and 5% clusters of 1000 cells, approximating findings from a recent autopsy study (17). For this analysis, we modeled different magnitudes of acinar tissue expansion within the affected region, ranging from a 20% to 100% increase in volume (a doubling of acinar tissue volume within the region). We found that 100% expansion was able to recapitulate the 90ml pancreas volume at 3% beta cell density in both the shell model and decay model (**Figure 4**). A 50% expansion of acinar cell volume reached a 90ml pancreas volume for the 30 µm shell thickness as well as 20 and 30 µm decay length. The lowest expansion magnitude, a 20% increase in acinar volume, only reached a pancreas volume of 90ml at 3% beta cell density in the decay model with the largest (30 µm) decay length.

## Discussion

This study models the relationship between peri-islet acinar tissue expansion seen on histology with reduced pancreas size observed in the diabetic pancreas. Using previously reported parameters for beta cell density and acinar cell expansion, we demonstrate that loss of acinar tissue surrounding islets is sufficient to explain the smaller pancreas observed in individuals with diabetes. Indeed, the model recapitulates autopsy studies demonstrating declines in pancreas size, beta cell number, and acinar cell number found in T1D (19). We further establish the range of islet sizes and extent of acinar tissue expansion that correspond with clinically observed changes on radiological imaging. Importantly, this model suggests that loss of individual beta cells and small beta cell clusters leads to larger declines in pancreas size than loss of large islets.

The model developed herein suggests that smaller clusters of beta cells have a greater influence on pancreas volume than larger clusters, due to reduced overlap in the volume of insulin action surround each cell. This finding has important implications for understanding changes in pancreas volume given the heterogenous range of islet sizes in the pancreas (13). Specifically, the loss of small islets may may cause more pancreas atrophy than large islets, on a per cell basis. This finding carries added significance given a number of recent reports demonstrating a preferential loss of small beta cell clusters in individuals with T1D, particularly in the early stages of the disease (17, 21–24). Our mathematical model suggests that the declines in pancreas size found using imaging may be inherently sensitive to this process. Specifically, our model predicts a linear relationship between pancreas size and the number of beta cells, but only for a given shell thickness/decay length and beta cell cluster size. As the slope of this linear relationship is dependent on these variables, temporal heterogeneity in beta cell loss, such as preferential loss of small endocrine clusters in early T1D, would lead to a non-linear relationship between beta cell mass and pancreas size over time. In this case, the pancreas volume would decline more quickly per beta cell lost in early T1D, with a slower decline in pancreas volume in later T1D as larger islets are lost. Beyond T1D, this model may also inform our understanding of other alterations in beta cell mass and/or pancreas size. Individuals with type 2 diabetes have a 50% deficit in beta cell mass (25) and a pancreas size intermediate between nondiabetic controls and T1D (1), in agreement with our model. In addition to understanding how declines in beta cell mass affect pancreas size, this model can simulate increases in beta cell mass/pancreas size, such as those observed in T2D remission (3).

Statistician George Box is credited with coining the aphorism ‘All models are wrong, but some are useful.’ This model is proposed in this spirit, as a first step toward modeling insulin action on acinar tissue, rather than a comprehensive model incorporating all aspects of insulin signaling and diabetes pathophysiology. As such, this mathematical model is limited by several simplifying assumptions. Our model assumed that individuals with T1D had complete loss of beta cell mass, but note that residual beta cells persist, even in longstanding T1D (26). This study modeled beta cells and their effect on surrounding acinar cells as spherical, but note that studies suggest that beta cells are elongated (14). Our model did not include other islet cells, either as drivers of acinar tissue expansion or contributors to cell cluster volume. For simplicity, we also assume the amount of insulin secreted by individual beta cells is independent of the size of the beta cell cluster. Importantly, insulin distribution is currently modeled as passive diffusion away from the beta cell; the model does not explicitly account for insulin consumption or binding to acinar cells (27). Finally, our model is unable to distinguish acinar cell hypertrophy from hyperplasia, the latter of which may be more dominant in humans (12).

Further studies are needed to both refine this model and validate it with histological studies. Model refinement includes incorporating the effects of other endocrine cells and the extracellular matrix, incorporating stochastic effects, and more sophisticated modeling of the insulin gradients surrounding islets. Importantly, our model assumes isotropic passive diffusion of insulin away from the beta cell. The vasculature surrounding the islet has complex structure that controls insulin delivery to acinar cells and modulates crosstalk between endocrine and exocrine cells (28). Future work may incorporate islet vasculature structure similar to our work modeling chemotherapy delivery to solid tumors (29). Our model is currently applied to one single point in time. However, it can be adapted to model temporal changes in beta cell mass during development of T1D and the effect of interventions that slow this process to better understand longitudinal imaging results.

In conclusion, the reduced pancreas size observed in individuals with T1D likely reflects not only beta cell loss but also acinar tissue atrophy in response to loss of insulin signaling. Radiological imaging can quantify the size of the pancreas, offering potential insights into disease progression or therapeutic response that may not be captured by blood-based measures alone. However, the relationship between pancreas size and beta cell mass remains incompletely understood. By integrating histological data on beta cell density and clustering from excised human pancreata, we developed a mathematical model that links structural changes in acinar and beta cell compartments to imaging-based measures of pancreas size. This framework provides a quantitative tool to interpret pancreas imaging, estimate relative beta cell loss, and bridge the gap between high-resolution autopsy studies and whole-organ radiological observations, advancing our understanding of pancreatic remodeling in diabetes.

## Data Availability

The original contributions presented in the study are included in the article/supplementary material. Further inquiries can be directed to the corresponding author.
